# Depressive symptoms associated with concerns about falling in Parkinson's disease

**DOI:** 10.1002/brb3.524

**Published:** 2016-07-21

**Authors:** Erika Franzén, David Conradsson, Maria Hagströmer, Maria H. Nilsson

**Affiliations:** ^1^ Division of Physiotherapy Department of NeurobiologyCare Sciences and SocietyKarolinska InstitutetHuddingeSweden; ^2^ Department of Physical TherapyKarolinska University HospitalSolnaSweden; ^3^ Department of Health SciencesLund UniversityLundSweden

**Keywords:** balance, depression, fear of falling, mobility devices, physical activity

## Abstract

**Background:**

Concerns about falling, a construct related to fear of falling, is increased in people with Parkinson's disease (PD) and is recognized as a barrier for exercise, negatively affecting health‐related quality of life and participation.

**Aim:**

To investigate modifiable factors associated with concerns about falling in elderly with mild‐to‐moderate PD.

**Methods:**

Eighty‐nine elderly (39 females, mean age 73 years) with mild‐to‐moderate PD were recruited. Concerns about falling were assessed with the Falls Efficacy Scale‐international, that is, the dependent variable in multiple linear regression analysis. Independent variables included both motor (e.g., objective measures of physical activity and gait) and nonmotor aspects such as depressive symptoms.

**Results:**

A model with three significant independent variables explained 33% of the variance in concerns about falling. According to the standardized regression coefficients (β), the strongest contributing factor was depressive symptoms (0.40), followed by balance performance (−0.25), and use of mobility devices (0.24).

**Conclusions:**

The findings imply that factors associated with concerns about falling are a multifactorial phenomenon. For its management in elderly with mild‐to‐moderate PD, one should consider depressive symptoms, balance deficits, and mobility devices.

## 1 Introduction

Fear of falling (FOF) is an umbrella term that covers fall‐related self‐efficacy, concerns about falling, balance confidence, and fall‐related activity avoidance (Jonasson, Nilsson, & Lexell, 2014; Kendrick et al., 2014). In elderly, several factors associated to FOF has been recognized, for example, a history of falls, female gender, physical function, walking aids, and depression or poor self‐related health (Denkinger, Lukas, Nikolaus, & Hauer, 2015; Chang, Chen, & Chou, 2016). In people with Parkinson's disease (PD), FOF is both more common as well as pronounced (Bloem, Grimbergen, Cramer, Willemsen, & Zwinderman, 2001) and has been recognized as a barrier for exercise, negatively affecting health‐related quality of life and participation (Rahman, Griffin, Quinn, & Jahanshahi, 2011; Ellis et al., 2013). Consequently, it is important to acknowledge FOF in health care and rehabilitation programs targeting people with PD. Interventions may impact directly on FOF or indirectly on factors associated with FOF, such as exercise interventions (Kendrick et al., 2014).

In order to develop interventions that aim at alleviating FOF, it is critical to determine which modifiable factors contribute to FOF in people with PD. Since FOF can be conceptualized in many ways, the different aspects of FOF may associate to diverse modifiable factors. Prior PD‐studies that used multivariate analyses have mainly targeted fall‐related self‐efficacy or balance confidence; the results indicate that walking difficulties and balance problems are of importance (Rahman et al., 2011; Mak, Pang, & Mok, 2012; Nilsson, Hariz, Iwarsson, & Hagell, 2012; Lindholm, Hagell, Hansson, & Nilsson, 2014). However, none of these studies included physical activity as an independent variable despite that it has been acknowledged to be a critical variable to address in relation to FOF (Denkinger et al., 2015). Furthermore, there is limited knowledge regarding contributing factors to concerns about falling (Jonasson, Ullén, Iwarsson, Lexell, & Nilsson, 2015), although this construct has shown advantages in assessing FOF in elderly and in PD (Jonasson et al., 2014). The aim of this study was therefore to explore modifiable factors associated with concerns about falling in elderly with mild‐to‐moderate PD.

## 2 Methods

### 2.1 Participants

This retrospective cross‐sectional study involved 89 community‐dwelling individuals with idiopathic PD (39 females, mean age 73 years, min‐max 61–87) who were recruited from an ongoing intervention study (BETA‐PD study, NCT01417598) (Conradsson, Löfgren, Ståhle, Hagströmer, & Franzén, 2012) and baseline data was used. All participants were independent ambulators (with or without a mobility device) and classified as Hoehn and Yahr stage 2 (42%) or 3 (58%). The mean duration of their disease was 5.8 years (min‐max 0.5–25), and they had an average levodopa equivalent daily dose of 612 mg (min‐max 0–2666) and all participants had a Mini Mental State Examination score above 24, mean 28 (min‐max 24–30). This study was approved by the regional board of ethics in Stockholm and all participants provided written informed consent.

### 2.2 Data collection

Data collection included variables that captured demographic information (age and gender), structured questions (history of falls in the last 12 months and use of mobility device using standardized questions administered as an interview), questionnaires (concerns about falling and depressive symptoms), clinical assessments (motor symptoms, gait, and balance performance), and objectively measured physical activity in daily life. The included instruments and means of assessment are clarified below.

Concerns about falling were assessed with the Falls Efficacy Scale‐international (FES‐I), which assesses concerns about falling during 16 daily activities, scored from 1 (not at all concerned) to 4 (very concerned), yielding a maximum score of 64 (higher score = worse) (Delbaere et al., 2010). The Geriatric Depression Scale 20 (GDS‐20) was used to assess depressive symptoms. It consists of 20 dichotomous (yes‐no) items; higher scores indicate more depressive symptoms.

Gait speed (min/s, comfortable pace) was assessed using an electronic walkway system, GAITRite^®^ (CIR Systems, Inc., Havertown, PA). The subjects were assessed walking six trials in their comfortable pace (approximately 50 steps in total). A 2.5 m deceleration and acceleration distance before and after the mat was used to ensure recording steady‐state walking. Motor symptoms were assessed according to the Unified PD Rating Scale (UPDRS) part III; the maximum total score is 108 points (higher = worse). Item 30 (pull test) of UPDRS III was used to assess reactive postural control in relation to an external perturbation in standing. In addition, balance performance was assessed with the Mini Balance Evaluation Systems Test (Mini‐BESTest), which consists of 14 items that cover different domains of balance control, scored from 0 (unable or requiring help) to 2 (normal); the maximum score is 28 points (higher = better) (Löfgren, Lenholm, Conradsson, Ståhle, & Franzén, 2014). Physical activity was assessed during free‐living conditions using accelerometers (Actigraph GT3X+, Pensacola, FL). Participants were instructed to wear the accelerometer on the waist for seven consecutive days and to record on a log sheet the exact times the device was worn. An average value of at least 4 days (each day with ≥9 hr of wear time) was used for analysis. Average steps per day was used as outcome variable which is strongly correlated with total physical activity (Harris et al., 2009; Tudor‐Locke et al., 2011).

### 2.3 Statistical analyses

Ten independent variables were considered for inclusion in the multivariate model (see Table 1). The following variables were dichotomized: history of falls, reactive postural control, and number of steps per day. History of falls data and number of steps per day were dichotomized since data were skewed. Therefore, we categorized these variables into recurrent fallers (i.e., >1 fall in the last 12 months) or nonfallers (i.e., one or no previous falls in the last 12 months) in accordance to ProFaNE (Lord, Sherrington, Menz, & Close, 2007) and <5000 steps per day or ≥5000 steps. Reactive postural control assessed with item 30 of the UPDRS part III was dichotomized as having normal postural reactions (0–1) or abnormal >2. After a logarithmic transformation of FES‐I scores (i.e., dependent variable), assumptions for parametric analyses were met. Data were checked for collinearity (*r* > 0.6) and univariate regression analyses were then employed for each variable. In order to avoid leaving a confounding variable out, all variables with a *p*‐value below 0.2 were entered into a multiple linear regression model and backward stepwise deletion was carried out. The final model was checked regarding underlying assumptions; the stability was checked by repeating the procedure using the forward method. The level of statistical significance was set to *p *< 0.05. Analyses were carried out using IBM SPSS Statistics, version 22.0.

**Table 1:** Sample characteristics and results of univariate regression analysis with FES‐I scores as the dependent variable, *n* = 89

Continuous variables	Total sample Mean (min‐max)	Univariate regression analysis *p*‐value
Age, years	73 (61–87)	0.762
Motor symptoms (UPDRS III)	37 (14–62)	0.110
Balance performance (Mini‐BESTest)	19 (12–25)	<0.001
Gait speed (GAITRite, min/s)	1.17 (0.67–1.53)	0.013
Depressive symptoms (GDS‐20)	4 (0–15)	<0.001

Dichotomous variables	Total sampleMean (min‐max)		Univariate regression analysis*p*‐value
	*n*/total	%	
Sex, woman	39/89	44	0.059
History of falls (>1 fall past year)	41/89	46	0.680
Reactive postural control (>2 item 30, UPDRS III)	54/89	61	0.013
Physical inactivity (<5000 steps/day)	55/89	62	0.044
Mobility devices, in‐ or/and outdoors^a^	39/89	44	<0.001

Possible score ranges: UPDRS III, 0–108 (higher = worse); Mini‐BESTest, 0–28 (higher = better); GDS‐20, 0–20 (higher = worse).

PD, Parkinson's disease; UPDRS, Unified Parkinson's Disease Rating Scale (part III = motor examination); GDS‐20, Geriatric Depression Scale.

a Of the 39/89 with mobility aids, 23 had walking sticks (Nordic walking poles), 6 cane, and 10 used a wheeled walker.

## 3 Results

FES‐I scores ranged from 17–63 (mean = 30, SD = 9.5). Eleven (12%) participants had low, 35 (39%) moderate, and 43 (48%) high concerns about falling (Delbaere et al., 2010).

Sample characteristics and univariate regression analysis are presented in Table 1.The results showed that age and history of falls were not significantly (*p* > 0.68) associated with concerns about falling. Eight independent variables (*p *< 0.2) were included in the multiple regression analysis, see footnote in Table 2. This resulted in a model with three significant independent variables, explaining 33% of the variance of FES‐I scores (Table 2). According to the standardized regression coefficients (β), the strongest independent variable was depressive symptoms, followed by balance performance and use of mobility devices.

**Table 2:** Multiple linear regression with concern about falling (FES‐I^a^) scores as the dependent variable among elderly with mild‐to‐moderate disease^b^, *n* = 89

Significant independent variables	B	95% CI	β	*p*‐value
Depressive symptoms	0.04	0.02, 0.05	0.40	<0.001
Mobility devices	0.14	0.03, 0.26	0.24	0.014
Balance performance	−0.02	−0.4, −0.01	−0.25	0.011

B, regression coefficient; β: standardized regression coefficient; CI, confidence interval; FES‐I, the Falls Efficacy Scale‐international; GDS‐20, Geriatric Depression Scale; UPDRS part III, Unified Parkinson's Disease Rating Scale (part III = motor examination).

a Logarithmic transformation of the FES‐I.

b Independent variables in the analysis were sex (1 = women), motor symptoms (UPDRS III), balance performance (Mini‐BESTest, higher scores = “better”), Postural instability (1 = instable on the Pull test, item 30 of UPDRS part III), Gait speed (min/s), mobility devices (1=yes), physical inactivity (<5000 steps/day = 1) and depressive symptoms (GDS‐20, higher scores = more depressive symptoms).

## 4 Discussion

These findings suggest that concern about falling is a multifactorial phenomenon and both motor (balance performance and use of mobility devices) and nonmotor symptoms (depression) may be of importance when addressing FOF in people with PD.

Although depressive symptoms have shown to be associated with perceived consequences of falling in people with PD, this finding has not been reported earlier for fall‐related self‐efficacy in this population (Rahman et al., 2011). Hence, this is the first study to shows that depressive symptoms independently contribute to concerns about falling in people with PD. This is supported by an earlier study investigating concerns about falling in PD where depressive symptoms were close to statistical significance in the final model (Jonasson et al., 2015). Furthermore, a systematic review in community‐dwelling older adults has shown that depressive symptoms are associated to activity avoidance but not to fall‐related self‐efficacy or balance confidence (Denkinger et al., 2015). In addition, depressive symptoms measured with the GDS have been shown to be associated with concerns about falling in elderly women with diabetes type 2 (Moreira Bde et al., 2016). The relationship between depressive symptoms and FOF is difficult to entangle and longitudinal studies are needed.

In this study, sex was not associated to FOF, which corroborates previous PD‐studies (Nilsson et al., 2012; Lindholm et al., 2014; Jonasson et al., 2015) but is in contrast to studies involving the general older population(Denkinger et al., 2015). Two other PD‐studies identified fatigue as an independent contributor to fall‐related self‐efficacy (Nilsson et al., 2012; Lindholm et al., 2014). The present finding corroborates that nonmotor symptoms seem to be of importance in relation to FOF.

Balance performance (Mini‐BESTest scores) assessed with a multiple item test addressing different aspects of balance control, was identified as a significant independent contributor to concerns about falling, whereas, reactive postural control (item 30, UPDRS III) did not independently contribute. This corroborates the findings by Lindholm et al., (2014), who found that functional balance performance (but not reactive postural control per se) contributed to fall‐related self‐efficacy. These findings suggest that, if targeting FOF, balance training should focus on exercises covering the complexity of balance performance in PD rather than only on reactive postural control in relation to an external perturbation.

The association between using a mobility device and concerns about falling may highlight the need of conducting follow‐ups on why such devices are used and how they are handled and perceived. Although this finding is in contrast to another study (Jonasson et al., 2015), the use of mobility devices could be seen as a proxy for walking difficulties. Walking difficulties, on the other hand has been identified as the strongest contributing factor in several previous studies (Nilsson et al., 2012; Lindholm et al., 2014; Jonasson et al., 2015). However, in line with a previous study (Lindholm et al., 2014), gait speed did not independently contribute to concerns about falling. Furthermore, in this study, the most common mobility devices were Nordic walking sticks (59%) or a cane (15%). Importantly, these devices are easily accessible without the involvement of health care professionals and could also be used as a training device, rather than for safety reasons or due to impaired balance control.

This study corroborates that a history of falls does not independently contribute to FOF in PD (Mak et al., 2012; Nilsson et al., 2012; Lindholm et al., 2014; Jonasson et al., 2015). FOF needs, thus, to be clinically addressed also among those who do not fall. However, it needs to be noted that FOF has been shown to predict future recurrent falls (Mak & Pang, 2009). That is, FOF seems to be a risk factor for future falls although a history of falls does not independently contribute to FOF.

Physical activity (measured objectively as steps per day) did not independently contribute to concerns about falling. This despite that physical activity levels have been shown to be low in a similar sample of elderly with PD (Benka Wallén, Franzén, Nero, & Hagströmer, 2015), and that reduced balance confidence has shown to independently explain self‐reported physical activity in people with PD (Bryant, Rintala, Hou, & Protas, 2015). This study is, in fact, the first study to include objective measures of physical activity as a potential explanatory factor to FOF in PD; future studies are therefore needed to confirm or refute our findings. It would also be of interest to study how objective measures of physical activity relates to different conceptualizations of FOF, that is, fall‐related self‐efficacy, concerns about falling, balance confidence, and fall‐related activity avoidance.

Some study limitations need to be acknowledged. The used convenience sample restricts the generalizability to younger people with PD or those with cognitive decline or more severe PD. In addition, the explanatory level of the final model was low (33%), which mirrors that many unaddressed aspects are of importance for concerns about falling in PD. Moreover, the cross‐sectional design does not allow for interpretation about cause‐and‐effect relationships. This highlights the need of longitudinal studies within this area that involves people with PD. The strengths of our study are that we used multivariate analysis including objective measures of gait as well as of physical activity.

These findings highlight that concerns about falling is a multifactorial phenomenon and that both motor and nonmotor symptoms may be of importance in people with PD. In fact, this study is the first to show that depressive symptoms contribute to concerns about falling in PD. These results have implications for the clinical management of concerns about falling in people with mild‐to‐moderate PD, by suggesting that one should pay attention to depressive symptoms, balance deficits, and mobility devices in rehabilitation programs of FOF.

## 5 Funding Information

This work was supported by Lund University (MultiPark), Swedish Parkinson Foundation, Karolinska Institutet (StratNeuro) , Swedish Research Council.

## Conflict of Interest

Authors have no conflicts of interest.
